# Comparative effectiveness of Cangrelor in patients with acute coronary syndrome undergoing percutaneous coronary intervention: an observational investigation from the M.O.Ca. registry

**DOI:** 10.1038/s41598-023-37084-2

**Published:** 2023-07-01

**Authors:** Martino Pepe, Eugenio Carulli, Claudio Larosa, Gianluigi Napoli, Palma Luisa Nestola, Maria Cristina Carella, Salvatore Giordano, Rocco Tritto, Francesco Bartolomucci, Plinio Cirillo, Giuseppe Biondi Zoccai, Arturo Giordano, Marco Matteo Ciccone

**Affiliations:** 1 Division of Cardiology, Department of Interdisciplinary Medicine (D.I.M.), University of Bari “Aldo Moro”, Bari, Italy; 2 Division of Cardiology, Lorenzo Bonomo Hospital, Andria, Italy; 3 Division of Cardiology, Department of Medical and Surgical Sciences, Magna Graecia University, Catanzaro, Italy; 4 Department of Advanced Biomedical Sciences, Federico II University of Naples, Naples, Italy; 5 Department of Medico-Surgical Sciences and Biotechnologies, Sapienza University of Rome, Latina, Italy; 6 Mediterranea Cardiocentro, Naples, Italy; 7 Invasive Cardiology Unit, “Pineta Grande” Hospital, Castel Volturno, Caserta Italy; 8 Cardiovascular Diseases Section, Cardiothoracic Department (DAI), University of Bari, Polyclinic of Bari, P.zza Giulio Cesare 11, 70124 Bari, Italy

**Keywords:** Cardiology, Medical research

## Abstract

Cangrelor, the first intravenous P2Y_12_ inhibitor (P2Y_12_-I), has been approved on the basis of three large RCTs from the CHAMPION program which nevertheless have been criticized for the low bleeding risk of the enrolled patients, the large quote of chronic coronary syndromes, and the use of Clopidogrel as control arm even in the setting of acute coronary syndromes (ACS). We sought to investigate, in the setting of ACS, the comparative performance of Cangrelor in terms of in-hospital ischemic and haemorrhagic outcomes compared with the current gold-standard of oral P2Y_12_-I. The study retrospectively enrolled 686 consecutive patients admitted to the Divisions of Cardiology of Policlinico of Bari and L. Bonomo Hospital of Andria for ACS and treated with percutaneous coronary intervention. The study population was divided according to the P2Y_12_-I treatment strategy in two groups: patients given an oral P2Y_12_-I and patients receiving Cangrelor in the cath lab followed by an oral P2Y_12_-I. Clinical endpoints included death, ischemic and bleeding events occurring during hospital stay. Cangrelor treated patients presented higher clinical risk profile at presentation and faced higher death rate. However, after PS matching, in-hospital mortality resulted comparable between the groups and Cangrelor use was associated with reduced in-hospital definite stent thrombosis (*p* = 0.03). Data from our real-world registry highlight that, in the setting of ACS, Cangrelor is prevalently used in patients with very challenging clinical presentations. The adjusted analysis provides for the first time promising data on stent thrombosis reduction associated with Cangrelor use.

## Introduction

Percutaneous coronary intervention (PCI) with drug eluting stent (DES) implantation has lately become the revascularization of choice for most patients with acute coronary syndromes (ACS)^1,2^. Despite the constant evolution of devices and techniques, stent thrombosis (ST) remains the clinically most relevant short-term complication after PCI, especially in the setting of ACS^3^. Full platelet inhibition is, indeed, required during percutaneous revascularizations and is achieved through dual antiplatelet therapy (DAPT): the association of acetylsalicylic acid and an inhibitor of the platelet P2Y_12_ receptor for adenosine 5’-diphosphate (P2Y_12_-I). Nevertheless, in time dependent clinical scenarios, effectiveness of DAPT is potentially hindered by the delayed effect derived by the oral administration of most P2Y_12_-I^4^. In fact, limitations of Clopidogrel, Prasugrel, and Ticagrelor (all oral P2Y_12_-I) are the slow onset and offset of action and the impossibility to be administrated or to be fully effective in patients with orotracheal intubation, vomit, and impaired intestinal absorption ^5^. In detail, the extremely short time gap between first medical contact (FMC) and primary PCI jeopardizes the effectiveness of the administration of oral P2Y_12_-I in ST elevation myocardial infarction (STEMI) patients, while in the setting of non-ST elevation (NSTE) ACS the administration of an oral P2Y_12_-I prior to coronary angiography (pretreatment strategy) is discouraged by the current ESC guidelines^1,6^.

In this scenario the potential role of Cangrelor, the first intravenous P2Y_12_-I approved by the European Medicines Agency (EMA) in 2017 based on the three large randomized clinical trials of the CHAMPION (Cangrelor versus standard therapy to achieve optimal management of platelet inhibition) program, is noteworthy^7–9^. However, these randomized trials have raised some criticisms such as the low bleeding risk of the enrolled cohorts, the large quote of chronic coronary syndromes (CCS), and mainly the use of Clopidogrel as control arm even in the setting of ACS. As Clopidogrel has not been the P2Y_12_-I of choice in ACS since 2012^10^, the latter limitation seems the most crucial and represents a confounder for the interpretation of data on both ST and bleeding. Aim of our study was to evaluate the real-world performance of Cangrelor in ACS patients in terms of in-hospital ischemic and hemorrhagic outcomes compared with the current gold-standard of oral P2Y_12_-I.

## Methods

The study, which was designed and written in accordance to the STROBE checklist, retrospectively enrolled all consecutive patients who accessed the Cardiology Divisions of the Azienda Ospedaliero Universitaria Consorziale—Policlinico of Bari and L. Bonomo Hospital of Andria with the diagnosis of ACS and underwent PCI. Enrollment started from the date of the first availability of Cangrelor in each center and ended in January 2021; the first patient treated with Cangrelor was in September 2019. The Independent Ethical Committee of the Azienda Ospedaliero Universitaria Consorziale Policlinico di Bari approved the study. Informed consent was obtained according to the study protocol. PCI procedures were performed per standard of care and at the discretion of the treating physicians. All treatments were carried out in accordance with current guidelines and regulations. The use of Cangrelor, which was administered only to P2Y_12_-I naïve patients, was decided by the interventional cardiologists on an individual basis, taking into consideration both clinical and procedural features. In all cases the time-point of Cangrelor administration was after coronary angiography and immediately before PCI with a 30 microg/kg bolus followed by a 4 microg/kg/min infusion as per label recommendations. The adjunctive pharmacological therapy was at physicians' discretion and largely based on contemporary best practice according to the national and European scientific societies' guidelines. Taking part to the study did not modify in any way patients’ diagnostic and therapeutic workup. The registry was broadly inclusive; the only exclusion criteria were age younger than 18 years and enrollment in other clinical trials. Information on demographics, baseline clinical characteristics, processes of care, and in-hospital outcomes were collected.

Due to the observatory nature of the study no preliminary hypotheses were generated. Clinical endpoints were evaluated during hospital stay and included death, ischemic and bleeding events. Bleeding was defined according to the Bleeding Academic Research Consortium (BARC), Global Use of Strategies to Open occluded coronary arteries (GUSTO), Thrombolysis in Myocardial Infarction (TIMI), and International Society on Thrombosis and Haemostasis (ISHT) definitions^11–14^, acute myocardial infarction (AMI) on the basis of its fourth universal definition^15^ and periprocedural myocardial infarction according to the CHAMPION PHOENIX definition^16^. Patients at high bleeding risk (HBR) were identified according to the Academic Research Consortium (ARC) definition^17^. The hemorrhagic risk was also calculated based on the PRECISE DAPT score^18^. Definite or probable ST was assessed according to the definition of the Academic Research Consortium^19^; in detail, definite ST was defined as symptoms suggestive of an acute coronary syndrome and angiographic or pathologic confirmation of stent thrombosis, while probable ST as an unexplained death within 30 days or target vessel myocardial infarction without angiographic confirmation of stent thrombosis. Complex PCI was defined as a procedure with at least one of the following angiographic characteristics: 3 vessels treated, ≥ 3 stents implanted, ≥ 3 lesions treated, bifurcation with deployment of 2 stents, total stent length > 60 mm, and chronic total occlusion^20,21^. High-risk clinical profile was defined as cardiogenic shock (CS) and/or treatment with inotropic drugs and/or cardiocirculatory arrest (CCA) and/or orotracheal intubation (OTI) at presentation. CS was defined as systolic blood pressure ≤ 90 mmHg (without inotropic drugs or intra-aortic balloon support) that is unresponsive to intravenous fluid administration, secondary to cardiac dysfunction, and associated with signs of hypoperfusion (cold extremities, impaired mental status, or urine output ≤ 30 ml/h)^22^.

The study population was divided according to the P2Y_12_-I treatment strategy in two groups: patients given an oral P2Y_12_-I and those who received Cangrelor in the cath lab followed by an oral P2Y_12_-I (non-Cangrelor and Cangrelor group respectively). Baseline characteristics, procedural features, and follow-up data of the overall population and per group are presented. All endpoints were assessed at the time of discharge or afterward and mean hospitalization time was 7.65 ± 5.50 and 7.07 ± 4.24 days for the Cangrelor and non-Cangrelor group respectively (*p* = 0.167).

The database was built up by Excel software (Microsoft Corporation, Redmond, Washington, USA); statistical analysis was performed using SPSS version 26 software (IBM, Inc., Armonk, NY). Continuous variables are presented as means ± standard deviations and compared using paired Student’s t-tests. Categorical variables are shown as numbers with percentages and analyzed using the chi-square analysis and Fisher’s exact test for counts < 5. The relationship between Cangrelor use and both baseline characteristics and procedural features was examined using univariate logistic regression analysis with odds ratio (OR) and 95% confidence intervals (CIs). Statistically significant (*p* < 0.05) predictors of Cangrelor use were entered into multivariable logistic regression models. The data underlying this article will be shared on reasonable request to the corresponding author.

For in-hospital mortality, the association with baseline characteristics, procedural features, and in-hospital adverse events has been tested with an univariate logistic regression analysis; ORs were calculated with 95% CIs. Each of the statistically significant (*p* < 0.05) predictor of outcome was entered into multivariable logistic regression models.

A propensity score (PS) analysis was also used to adjust for differences in patients’ baseline and procedural characteristics; the following parameters were selected: age, gender, diabetes mellitus (DM), STEMI diagnosis, chronic kidney disease (CKD), high-risk clinical profile, HBR profile, left ventricle ejection fraction (LVEF) < 30%, and femoral access. These covariates were chosen among those significantly different within our population between the Cangrelor and non-Cangrelor group and/or significantly associated with mortality in the multivariate logistic regression model and/or well-known predictors of adverse events from the literature. The 1:1 nearest neighbor matching without replacement method was used (standard deviation and caliper value were 0.11 and 0.2 respectively) and performed by PScore module from Statistics for Data Analysis powered by SPSS. Standardized differences and c-statistic were used to confirm negligible differences in the mean or prevalence of selected covariates between treatment groups. For all tests significance was set for a 2-tailed value of *p* < 0.05.

## Results

Cangrelor group and non-Cangrelor group included 198 and 488 patients, respectively. Mean age of the whole population was 67.4 ± 11.7 years; baseline clinical characteristics of patients as a whole and by group are depicted in Table 1. Patients in the non-Cangrelor group showed higher prevalence of DM and of prior AMI, PCI, and myocardial revascularization. Conversely, Cangrelor group presented higher-risk clinical profile confirmed by greater prevalence of LVEF < 30%, inotropic drug infusion, CCA, CS, and previous haemorrhages. Supplementary Table 1 shows oral P2Y_12_-I treatment in the overall population and by group: clopidogrel use was more prevalent in the Cangrelor group.

**Table 1:** Baseline characteristics of the overall population and by groups.

	Overall (n = 686)	Cangrelor (n = 198)	Non cangrelor (n = 488)	*p*
Age, yrs	67.42 ± 11.69	68.62 ± 11.11	66.95 ± 11.90	0.090
Male sex	535 (78.0%)	149 (75.3%)	386 (79.1%)	0.271
STEMI	363 (52.9%)	115 (58.1)	248 (50.8%)	0.084
Diabetes mellitus	169 (24.6%)	38 (19.2%)	131 (26.8%)	0.035
Arterial hypertension	522 (76.1%)	155 (78.3%)	367 (75.2%)	0.392
Dyslipidaemia	432 (63.0%)	135 (68.2%)	297 (60.9%)	0.072
Current smoker	227 (33.1%)	66 (33.3%)	161 (33.0%)	0.931
Family history of CAD	104 (15.2%)	43 (21.7%)	61 (12.5%)	0.002
Obesity	129 (18.8%)	37 (18.7%)	92 (18.9%)	0.960
Prior percutaneous coronary intervention	139 (20.3%)	29 (14.6%)	110 (22.5%)	0.020
Prior coronary bypass	48 (7.0%)	10 (5.1%)	38 (7.8%)	0.203
Prior myocardial revascularization	161 (23.5%)	36 (18.2%)	125 (25.6%)	0.037
Prior myocardial infarction	125 (18.2%)	22 (11.1%)	103 (21.1%)	0.002
Prior stroke	13 (1.9%)	2 (1.0%)	11 (2.3%)	0.367
Prior haemorrhages	9 (1.3%)	6 (3.0%)	3 (0.6%)	0.020
Peripheral artery disease	49 (7.1%)	16 (8.1%)	33 (6.8%)	0.543
Recent major trauma or surgery	18 (2.6%)	3 (1.5%)	15 (3.1%)	0.302
Chronic kidney disease	144 (21.0%)	47 (23.7%)	97 (19.9%)	0.261
Chronic OAC therapy	61 (8.9%)	17 (8.6%)	44 (9.0%)	0.858
eGFR	80.52 ± 27.88	81.03 ± 32.53	80.32 ± 25.88	0.773
Creatinine	1.08 ± 0.79	1.08 ± 0.83	1.07 ± 0.78	0.914
Glycemia	132.82 ± 60.06	134.36 ± 67.26	132.23 ± 57.11	0.685
LDL	103.82 ± 39.91	103.03 ± 38.46	104.10 ± 40.45	0.776
Haemoglobin (g/dL)	13.48 ± 2.01	13.49 ± 2.02	13.48 ± 2.01	0.965
Platelets (/mmc)	231.82 ± 83.41	227.33 ± 86.30	233.62 ± 82.25	0.383
White blood cells (^10^3^/mmc)	10.5 ± 4.1	10.6 ± 4.2	10.5 ± 4.0	0.841
LVEF at admission (%)	46.62 ± 9.08	46.18 ± 10.19	46.79 ± 8.61	0.451
LVEF ≤ 30%	62 (9.0%)	34 (17.2%)	28 (5.7%)	< 0.001
Non-invasive ventilation	30 (4.4%)	9 (4.5%)	21 (4.3%)	0.897
High-risk clinical profile	102 (14.9%)	41 (20.7%)	61 (12.5%)	0.006
HBR-ARC	229 (33.4%)	63 (31.8%)	166 (34.0%)	0.580
PRECISE DAPT ≥ 25	223/646 (34.7%)	72 (40.4%)	151 (32.5%)	0.060
Inotropic drugs infusion	65 (9.5%)	29 (14.6%)	36 (7.4%)	0.003
Orotracheal intubation	59 (8.6%)	23 (11.6%)	36 (7.4%)	0.075
Cardiocirculatory arrest	61 (8.9%)	25 (12.6%)	36 (7.4%)	0.030
Shock	75 (11.0%)	29 (14.6%)	46 (9.5%)	0.049

Values are expressed as mean ± SD or n (%).

*STEMI* ST-elevation myocardial infarction, *CAD* Coronary artery disease, *OAC* Oral anticoagulation, *eGFR* Estimated glomerular filtration rate, *LDL* Low-density lipoprotein, *LVEF* Left ventricular ejection fraction, *HBR-ARC* High bleeding risk according to Academic Research Consortium.

In the univariate and multivariate logistic regression analysis, predictors of Cangrelor use resulted prior bleeding and LVEF < 30%; high-risk clinical profile reached threshold for significance in the univariate while only approached significance in the multivariate analysis (Table 2).

**Table 2:** Association between Cangrelor use and baseline/procedural features.

	Univariate logistic regression analysis				Multivariate logistic regression analysis			
	95% C.I		OR	*p*	95% C.I		OR	*p*
Age	0.998	1.027	1.012	0.090				
Female	0.843	1.837	1.245	0.271				
STEMI	0.961	1.872	1.341	0.085				
Diabetes mellitus	0.431	0.972	0.647	**0.036**	0.464	1.099	0.714	0.126
Arterial hypertension	0.800	1.765	1.188	0.392				
Dyslipidaemia	0.971	1.956	1.378	0.073				
Smoking	0.715	1.442	1.016	0.931				
Obesity	0.648	1.510	0.989	0.960				
Prior PCI/coronary bypass	0.422	0.966	0.638	**0.034**	0.767	2.975	1.511	0.232
Prior AMI	0.285	0.765	0.467	**0.003**	0.144	0.708	0.319	**0.005**
Prior Bleeding	1.251	20.404	5.052	**0.023**	1.314	24.175	5.636	**0.020**
Peripheral artery disease	0.651	2.256	1.212	0.544				
OAC use	0.528	1.703	0.948	0.858				
CKD	0.845	1.864	1.255	0.261				
LVEF < 30%	2.003	5.792	3.406	** < 0.001**	1.968	6.102	3.465	** < 0.001**
High-risk clinical profile	1.182	2.827	1.828	**0.007**	0.934	2.388	1.494	0.094
NIV	0.476	2.354	1.059	0.888				
LM PCI	0.385	3.276	1.123	0.831				
LAD PCI	0.805	1.559	1.120	0.500				
CX PCI	0.440	1.028	0.672	0.067				
RC PCI	0.718	1.474	1.028	0.878				
Multivessel CAD	0.437	0.852	0.610	**0.004**	0.459	0.927	0.653	**0.017**
Complex PCI	0.785	1.826	1.197	0.404				
Multivessel PCI	0.849	2.240	1.379	0.194				
HBR-ARC	0.636	1.288	0.905	0.580				

*STEMI* ST-elevation myocardial infarction, *pci* percutaneous coronary intervention, *AMI* Acute myocardial infarction, *OAC* Oral anticoagulation, *CKD* Chronic kidney disease, *LVEF* Left ventricular ejection fraction, *NIV* Non invasive ventilation, *LM* Left main, *LAD* Left anterior descending coronary artery, *Cx* Circumflex coronary artery, *RC* Right coronary artery, *CAD* Coronary artery disease, *HBR-ARC* High bleeding risk according to Academic Research Consortium.

Significant values are in [bold].

Procedural features and in hospital follow-up data are described in Tables 3 and 4 respectively. Cangrelor group showed higher rate of femoral access and higher stent number and total stent length, despite a lower quote of multivessel coronary artery disease (CAD). In terms of clinical outcomes, Cangrelor treated patients faced higher occurrence of all-cause death. In the univariate logistic regression analysis, age, female sex, STEMI presentation, DM, CKD, LVEF < 30%, high-risk clinical profile, non-invasive ventilation (NIV), complex PCI, multivessel PCI, left-main (LM) PCI, femoral access, HBR profile, Cangrelor use, and in-hospital bleeding were associated with in-hospital all-cause death. The multivariate analysis proved that only age, STEMI, high-risk clinical profile, femoral access, and in-hospital bleeding were associated with in-hospital mortality (Table 5).

**Table 3:** Procedural features of the overall population and by groups.

	Overall (n = 686)	Cangrelor (n = 198)	Non cangrelor (n = 488)	*p*
Femoral access	113 (16.5%)	44 (22.2%)	69 (14.1%)	0.010
Multivessel CAD	412 (60.1%)	102 (51.5%)	310 (63.5%)	0.004
Treated vessel				
LAD	343 (50.0%)	103 (52.0%)	240 (49.2%)	0.500
CX	149 (21.7%)	34 (17.2%)	115 (23.6%)	0.066
RCA	205 (29.9%)	60 (30.3%)	145 (29.7%)	0.878
LM	16 (2.3%)	5 (2.5%)	11 (2.3%)	0.831
SVG	10 (1.5%)	2 (1.0%)	8 (1.6%)	0.732
Stent number/pt	1.37 ± 0.75	1.47 ± 0.81	1.32 ± 0.72	0.021
Stent number ≥ 2	215 (31.3%)	71 (35.9%)	144 (29.5%)	0.104
Total stent length	35.84 ± 20.65	38.94 ± 23.60	34.50 ± 19.12	0.012
Multivessel PCI	83 (12.1%)	29 (14.6%)	54 (11.1%)	0.193
Bifurcations	76 (11.1%)	27 (13.6%)	49 (10.0%)	0.174
IIb/IIIa inhibitors infusion	21 (3.1%)	3 (1.5%)	18 (3.7%)	0.219
Drug eluting balloon	38 (5.5%)	10 (5.1%)	28 (5.7%)	0.721
Complex PCI*	122 (17.8%)	39 (19.7%)	83 (17.0%)	0.404
≥ 3 lesions	13 (1.9%)	3 (1.5%)	10 (2.0%)	0.642
≥ 3 vessels	7 (1.0%)	4 (2.0%)	3 (0.6%)	0.111
≥ 3 stents	50 (7.3%)	21 (10.6%)	29 (5.9%)	0.033
≥ 60 mm total stent length	88 (12.8%)	34 (17.2%)	54 (11.1%)	0.030
2-stents technique bifurcations	31 (4.5%)	8 (4.0%)	23 (4.7%)	0.701
Chronic total occlusion lesions	4 (0.6%)	0 (0%)	4 (0.8%)	0.583
Transferred for surgical revascularization	28 (4.1%)	8 (4.0%)	20 (4.1%)	0.972
Slow/no reflow	24 (3.5%)	6 (3.0%)	18 (3.7%)	0.671

Values are expressed as mean ± SD or n (%).

*CAD* Coronary artery disease, *LAD* Left anterior descending coronary artery, *CX* Circumflex coronary artery, *RC* Right coronary artery, *LM* Left main, *SVG* Saphenous vein graft, *PCI* Percutaneous coronary intervention, *SVG* Simple venous graft, *PCI* Percutaneous coronary intervention.

*See text for definition.

**Table 4:** In-hospital follow-up data of the overall population and by groups.

	Overall		Cangrelor		Non cangrelor		*p*
	n = 686	(%)	n = 198	(%)	n = 488	(%)	
Contrast induced nephropathy	20	(2.9)	7	(3.5)	13	(2.7)	0.539
All-cause death	48	(7)	24	(12.1)	24	(4.9)	**0.001**
Any bleedings	16	(2.3)	5	(2.5)	11	(2.3)	0.511
Any ischemic cerebro-cardiovascular complications*	26	(3.8)	4	(2)	22	(4.5)	0.122
Myocardial infarction	20	(2.9)	4	(2)	16	(3.3)	0.375
Periprocedural myocardial infarction	16	(2.3)	3	(1.5)	13	(2.7)	0.275
Definite/probable stent thrombosis	11	(1.6)	2	(1)	9	(1.8)	0.341
Definite stent thrombosis	10	(1.5)	1	(0.5)	9	(1.8)	0.166
Probable stent thrombosis	1	(0.1)	1	(0.5)	0	(0)	0.289
BARC bleeding ≥ 3	14	(2)	5	(2.5)	9	(1.8)	0.378
TIMI major bleeding	3	(0.4)	2	(1)	1	(0.2)	0.201
TIMI at least minor bleeding	10	(1.5)	5	(2.5)	5	(1)	0.130
ISTH major bleeding	12	(1.7)	4	(2)	8	(1.6)	0.473
GUSTO severe bleeding	1	(0.1)	1	(0.5)	0	(0)	0.289
GUSTO at least moderate bleeding	12	(1.7)	5	(2.5)	7	(1.4)	0.245

Values are expressed as mean ± SD or n (%).

*PCI* Percutaneous coronary intervention,

*Acute myocardial infarction, probable/definite ST, TIA/stroke.

Significant values are in [bold].

**Table 5:** Association between in-hospital mortality and baseline characteristics, procedural features, and hemorrhagic and thrombotic in-hospital complications.

	Univariate logistic regression analysis				Multivariate logistic regression analysis			
	95% C.I		OR	*p*	95% C.I		OR	*p*
Age	1.036	1.099	1.067	** < 0.001**	1.027	1.134	1.079	**0.002**
Female	1.108	3.840	2.063	**0.022**	0.228	1.422	0.569	0.227
STEMI	2.898	16.490	6.913	** < 0.001**	2.476	44.302	10.473	**0.001**
Diabetes mellitus	1.049	3.569	1.935	**0.035**	0.760	5.618	2.066	0.155
Arterial hypertension	0.476	1.849	0.938	0.854				
Dyslipidaemia	0.342	1.112	0.617	0.108				
Smoking	0.176	0.832	0.383	**0.015**	0.127	1.242	0.398	0.113
Obesity	0.390	1.873	0.855	0.695				
Prior PCI/coronary bypass	0.628	2.364	1.219	0.558				
Prior AMI	0.579	2.472	1.197	0.627				
Peripheral artery disease	0.129	2.323	0.547	0.413				
History of bleeding	0.205	13.681	1.676	0.630				
OAC use	0.460	3.175	1.208	0.701				
CKD	1.962	6.528	3.579	** < 0.001**	0.735	5.559	2.022	0.173
LVEF < 30%	2.543	10.075	5.061	** < 0.001**	0.859	6.950	2.443	0.094
High-risk clinical profile	18.311	85.424	39.550	** < 0.001**	9.264	70.566	25.568	** < 0.001**
NIV	2.346	13.369	5.600	** < 0.001**	0.221	3.082	0.825	0.774
Multivessel CAD	0.730	2.526	1.358	0.334				
Complex PCI	1.486	5.213	2.784	**0.001**	0.655	4.841	1.781	0.258
Multivessel PCI	1.142	4.784	2.337	**0.020**	0.674	8.287	2.364	0.179
LM PCI	3.111	25.875	8.971	** < 0.001**	0.154	5.002	0.878	0.884
Femoral access	7.127	25.804	13.561	** < 0.001**	2.110	11.939	5.020	** < 0.001**
Slow/no reflow	0.920	8.577	2.809	0.070				
HBR-ARC	1.282	4.173	2.312	**0.005**	0.190	1.531	0.539	0.246
Cangrelor use	1.475	4.820	2.667	**0.001**	0.609	3.749	1.511	0.374
In-hospital bleedings	1.469	15.313	4.742	**0.009**	2.229	68.000	12.312	**0.004**
In-hospital ischemic complications*	0.840	7.711	2.545	0.099				

*STEMI* ST-elevation myocardial infarction, *PCI* Percutaneous coronary intervention, *AMI* Acute myocardial infarction, *OAC* Oral anticoagulation, *CKD* Chronic kidney disease, *LVEF* Left ventricular ejection fraction, *NIV* Non invasive ventilation, *CAD* Coronary artery disease, *LM* Left main, *HBR-ARC* High bleeding risk according to Academic Research Consortium.

*Acute myocardial infarction, probable/definite ST, TIA/stroke.

Significant values are in [bold].

**Figure 1:**
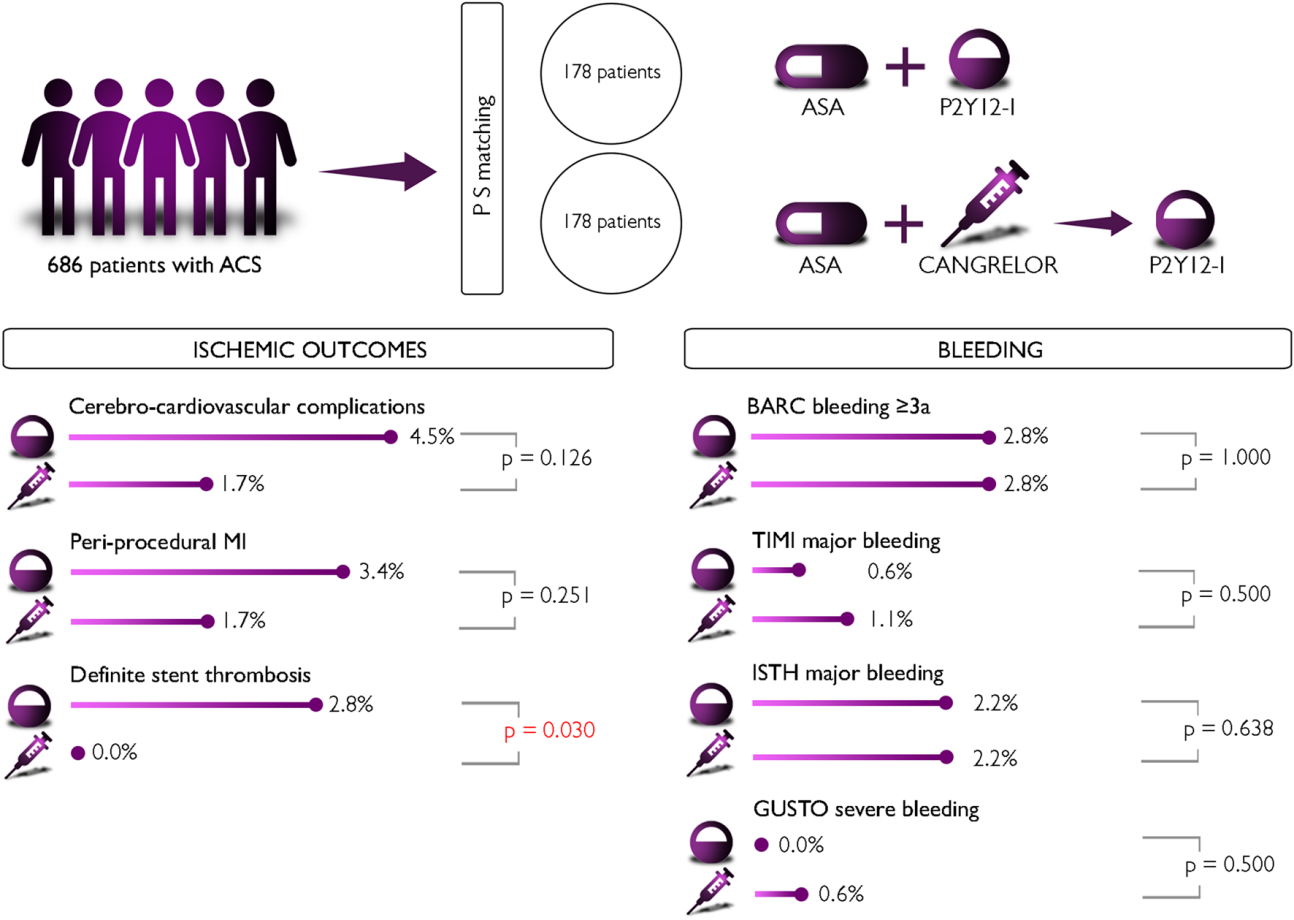
*ACS* Acute coronary syndrome, *PS* Propensity score, *ASA* Acetylsalicylic acid; P2Y_12_-I, P2Y_12_ inhibitor; *MI* Myocardial infarction, *BARC* Bleeding Academic Research Consortium, *GUSTO* Global use of strategies to open occluded coronary arteries, *TIMI* Thrombolysis in myocardial infarction, *ISTH* International Society on Thrombosis and Haemostasis.

After PS-matching a population of 356 patients was selected; baseline clinical characteristics are shown in Supplementary Table 2. C-statistic, used as post-matching diagnostic, and standardized differences confirmed negligible differences in the mean or prevalence of the selected covariates (age, gender, DM, STEMI, CKD, high-risk clinical profile, HBR-ARC, LVEF < 30%, and femoral access) between treatment groups (Supplementary Fig. 1). Table 6 summarizes the in-hospital follow-up data of the PS-matched population: noteworthy, no statistically significant difference between the two groups was found in terms of all-cause death. Nonetheless, in divergence with the results of the unmatched population, Cangrelor use was associated with reduced in-hospital definite stent thrombosis (*p* = 0.03) (Fig. 1).

**Table 6:** In-hospital follow-up data in the propensity-score matched (PSM) population.

	PSM population		Cangrelor		Non Cangrelor		*p*
	n = 356	(%)	n = 178	(%)	n = 178	(%)	
Contrast induced nephropathy	10	(2.8)	7	(3.9)	3	(1.7)	0.168
All-cause death	26	(7.3)	17	(9.6)	9	(5.1)	0.103
Any bleedings	11	(3.1)	5	(2.8)	6	(3.4)	0.759
Any ischemic cerebro-cardiovascular complications*	11	(3.1)	3	(1.7)	8	(4.5)	0.126
Myocardial infarction	10	(2.8)	3	(1.7)	7	(3.9)	0.168
Periprocedural myocardial infarction	9	(2.5)	3	(1.7)	6	(3.4)	0.251
Definite/probable stent thrombosis	6	(1.7)	1	(0.6)	5	(2.8)	0.107
Definite stent thrombosis	5	(1.4)	0	(0.0)	5	(2.8)	**0.030**
Probable stent thrombosis	1	(0.3)	1	(0.6)	0	(0)	0.500
BARC bleeding ≥ 3a	10	(2.8)	5	(2.8)	5	(2.8)	1.000
TIMI major bleeding	3	(0.8)	2	(1.1)	1	(0.6)	0.500
TIMI at least minor bleeding	7	(2)	5	(2.8)	2	(1.1)	0.224
ISTH major bleeding	8	(2.2)	4	(2.2)	4	(2.2)	0.638
GUSTO severe bleeding	1	(0.3)	1	(0.6)	0	(0)	0.500
GUSTO at least moderate bleeding	9	(2.5)	5	(2.8)	4	(2.2)	0.500

Values are expressed as mean ± SD or n (%).

*PCI* Percutaneous coronary intervention, *PSM* Propensity-score matched.

*Acute myocardial infarction, probable/definite ST, TIA/stroke.

Significant values are in [bold].

## Discussion

The main findings of our paper are the following: 1. Cangrelor was mainly used in ACS patients with high-risk clinical features and tendency to high bleeding risk; 2. the Cangrelor group underwent more extensive and complex coronary revascularization; 3. the Cangrelor group faced higher in-hospital mortality, which turned to be comparable between the two groups after PS adjustment for baseline clinical risk profile; 4. in the adjusted analysis Cangrelor use was associated with reduced in-hospital definite stent thrombosis in the absence of increased bleeding complications.

The present study explored the use of Cangrelor in the clinical scenario of ACS patients treated with PCI. To the best of authors’ knowledge this is the first real world investigation which analyzed in a comparative fashion Cangrelor performance. Our data confirmed that Cangrelor is more often used in clinically unstable patients such as those with CS and/or treated with inotropic drugs and/or with CCA at presentation and/or intubated and, concordantly, in those with a severely reduced LVEF. This is in line with previous evidence^23,24^ and can be partly explained by the impracticability of the oral route or the uncertainty of intestinal absorption in patients with high-risk clinical presentation, both limitations easily overcome by the intravenous administration of Cangrelor. Moreover, our analysis suggests the possible preference towards Cangrelor in patients with higher risk of bleeding as indicated by the higher rate of patients with history of previous bleeding and the higher (despite at the limit for significance) PRECISE DAPT score in the Cangrelor group. The higher bleeding risk in the Cangrelor group is further indirectly supported by the wider use of Clopidogrel in this group, which cannot be explained by the need for triple antithrombotic therapy being the prevalence of oral anticoagulation comparable between the two groups. This therapeutic choice could be hypothesized to be founded upon the rapid pharmacokinetic, in this case the fast offset of action, of Cangrelor which is likely perceived by the interventional cardiologists to be safer and more manageable than oral P2Y_12_-I.

In addition, procedural data highlight that, despite a greater quote of patients with multivessel CAD in the non-Cangrelor group, the patients treated with Cangrelor underwent more complex percutaneous interventions with a higher number of implanted stents per patient and a higher total stent length. Given the observational nature of the study, it can be only assumed that interventional cardiologists feel more confident in performing more extensive revascularizations when a full and rapid antiaggregation is guaranteed by the use of this intravenous antiplatelet agent.

Prerogative of Cangrelor, as mentioned above, is the rapidity of both onset and offset of action. Pharmacokinetic studies have proved indeed that platelet function is completely restored within 60 min after the stop of drug infusion, and Cangrelor is accordingly considered a periprocedural drug. Based on this assumption, and in line with the CHAMPION studies, the rationale for a clinical follow-up exceeding the hospital stay is lacking. Our outcome data suggest a trend toward better ischemic outcomes (lower rates of ischemic cerebro-cardiovascular complications, periprocedural AMI, definite ST) and slightly worse hemorrhagic complications. Despite the sample size does allow only hypotheses, these results appear in line with the registration trials of the CHAMPION program^7–9^. Notwithstanding the randomized nature, the CHAMPION studies present some limitations which have been widely recognized over time. Firstly the CHAMPION population was at relatively low ischemic risk since more than 30% of patients were addressed to PCI because of CCS^25^, which does not reflect the prevalent clinical setting in which the drug has been used, so far, in the real-world as suggested by several recent registries^23,24,26^. The second and probably the main point of criticism against the CHAMPION studies is the use of Clopidogrel in the control arm, despite more than two third of patients had ACS. In this subpopulation the reliability of the comparative evaluation of Cangrelor performance in terms of both ischemic and hemorrhagic events could result jeopardized. In opposition, in our study almost 80% of patients in the non-Cangrelor group received either Ticagrelor or Prasugrel (74.6% and 5.1% respectively) in line with the contemporary guidelines’ recommendations^1,2^.

On the other hand, our data must be interpreted with caution because of the non randomized nature of the enrollment. The Cangrelor group faced a significantly higher mortality because of the propensity to use this “new therapeutic weapon”, which allows to avoid bowel absorption and provides roughly instantaneous antiplatelet effect, in the most critical clinical scenarios. When we searched for the determinants of in-hospital death, age, high-risk clinical profile, in-hospital bleeding, and STEMI presentation resulted indeed predictors of outcome, while Cangrelor use did not.

Purposively, discrepancies in baseline clinical risk profile have been overcome with the propensity score matching. The adjusted analysis highlighted the absence of significant differences between groups in terms of mortality, which confirms our previous assumption. Even more remarkable, we found a significant reduction of in-hospital definite ST in the Cangrelor group, which was the key secondary endpoint of the Champion Phoenix trial. To the best of authors' knowledge, this finding represents the first report of reduced ST with Cangrelor in comparison to a group prevalently treated with the most potent oral P2Y_12_-I Ticagrelor and Prasugrel. Noteworthy, at variance with the registration trials, our endpoints were evaluated during hospital stay and not at 48 h from PCI; as a consequence we cannot exclude the influence of the oral P2Y_12_-I the Cangrelor patients have been switched into after infusion. Nevertheless, the Cangrelor group showed a higher percentage of patients treated with Clopidogrel than the non-Cangrelor group and this evidence further substantiates Cangrelor efficacy in preventing ST. In the matched population the use of Cangrelor did not conversely result to be associated to higher rate of bleeding events.

The present study should be interpreted in the light of some limitations. First, this was a nonrandomized study resulting in cohorts with differences in baseline, angiographic, and procedural characteristics. Although we sought to reduce potential confounding using PS-matching analysis, we were not able to correct for the unmeasured variables. Second, the use of Cangrelor and the entire procedural strategy were at the discretion of the physician. Third, sample size is small. As a consequence, our findings should be regarded as only hypotheses generating and would require further confirmation from a large, pragmatic, and randomized trial. Nevertheless, it is authors’ opinion that randomized trials on ACS patients treated with Cangrelor are not expected.

## Conclusion

Data from our real-world registry highlight that in the ACS context Cangrelor is prevalently used in patients with very challenging clinical presentations. This bias justifies the higher mortality rate in the Cangrelor group at the unadjusted analysis. On the other hand, the adjusted analysis suggests the potential replicability in the real world of the beneficial effect of Cangrelor in terms of definite ST suggested by the randomized trials, what’s more in a population treated according to the current gold-standard of antithrombotic therapy.

## Supplementary Information

Supplementary Figures.

Supplementary Tables.

## Data Availability

The data underlying this article will be shared on reasonable request to the corresponding author.
