# Supplementary material for: Comparative effectiveness of Cangrelor in patients with acute coronary syndrome undergoing percutaneous coronary intervention: an observational investigation from the M.O.Ca. registry
Source: Sci Rep. 2023 Jul 1;13:10685. doi: 10.1038/s41598-023-37084-2 (PMC10314899; doi:10.1038/s41598-023-37084-2)
Supplement: Supplementary file 1 — Supplementary Figures. [file 41598_2023_37084_MOESM1_ESM.docx]

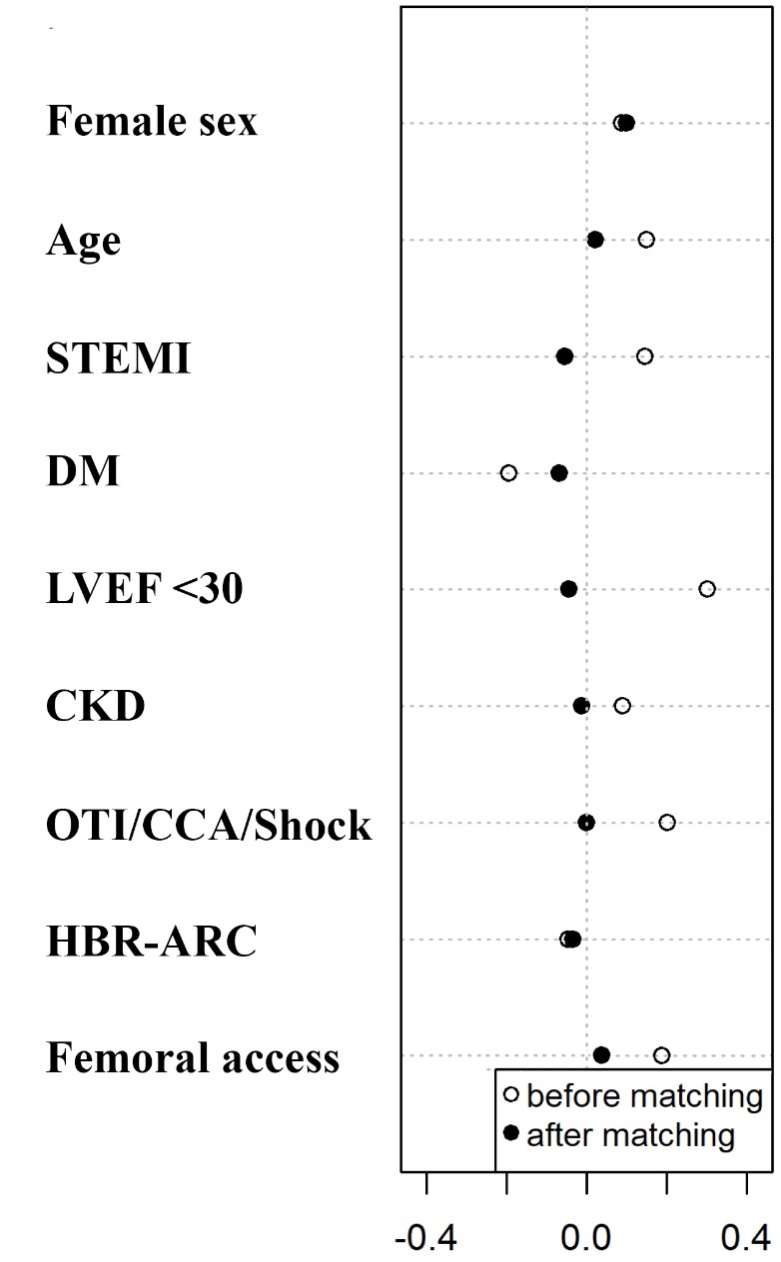
**Supplementary Figure 1.** Covariate balance plot before and after propensity matching, standardized for comparability.

STEMI, ST-elevation myocardial infarction; DM, diabetes mellitus; LVEF, left ventricle ejection fraction; CKD, chronic kidney disease; OTI, orotracheal intubation; CA, cardiociculatory arrest; HBR-ARC, High Bleeding Risk profile as defined by Academic Research Consortium.
