# Supplementary material for: Comparative effectiveness of Cangrelor in patients with acute coronary syndrome undergoing percutaneous coronary intervention: an observational investigation from the M.O.Ca. registry
Source: Sci Rep. 2023 Jul 1;13:10685. doi: 10.1038/s41598-023-37084-2 (PMC10314899; doi:10.1038/s41598-023-37084-2)
Supplement: Supplementary file 2 — Supplementary Tables. [file 41598_2023_37084_MOESM2_ESM.docx]

***Table S1. P2Y_12_ Inhibitor and triple antithrombotic therapy in the overall population and by group.***

|  | ***Overall***  *(n=686)* | ***Non Cangrelor***  *(n=488)* | ***Cangrelor***  *(n=198)* | ***P*** |
| --- | --- | --- | --- | --- |
| Ticagrelor | 481 (70.1%) | 364 (74.6%) | 117 (59.1%) | <0.001 |
| Prasugrel | 48 (7.0%) | 25 (5.1%) | 23 (11.6%) | 0.003 |
| Clopidogrel | 157 (22.9%) | 99 (20.3%) | 58 (29.3%) | 0.011 |
| Triple antithrombotic therapy | 81 (11.8%) | 27 (13.6%) | 54 (11.1%) | 0.344 |

Values are expressed as n (%).

***Table S2. Baseline characteristics of the PS matched population***

|  | ***Overall***  *(n=356)* |  | ***Cangrelor***  *(n=178)* |  | ***Non Cangrelor***  *(n=178)* |  | ***P*** |
| --- | --- | --- | --- | --- | --- | --- | --- |
| Age >75 | 108 | (30.3) | 52 | (29.2) | 56 | (31.5) | 0.645 |
| Age | 68.2 | ±11.5 | 68.3 | ±11.2 | 68.2 | ±11.7 | 0.846 |
| Male sex | 276 | (77.5) | 136 | (76.4) | 140 | (78.7) | 0.612 |
| STEMI | 201 | (56.5) | 100 | (56.2) | 101 | (56.7) | 0.915 |
| DiabetesMellitus | 75 | (21.1) | 38 | (21.3) | 37 | (20.8) | 0.897 |
| ArterialIperthension | 274 | (77.0) | 140 | (78.7) | 134 | (75.3) | 0.450 |
| Dislypidemia | 236 | (66.3) | 124 | (69.7) | 112 | (62.9) | 0.175 |
| Smoker status | 118 | (33.1) | 62 | (34.8) | 56 | (31.5) | 0.499 |
| Familiar history of CAD | 64 | (18.0) | 35 | (19.7) | 29 | (16.3) | 0.408 |
| Obesity | 65 | (18.3) | 35 | (19.7) | 30 | (16.9) | 0.493 |
| Previous PCI | 62 | (17.4) | 25 | (14.0) | 37 | (20.8) | 0.094 |
| Previous CABG | 24 | (6.7) | 10 | (5.6) | 14 | (7.9) | 0.398 |
| Priorrevascularization | 75 | (21.1) | 32 | (18.0) | 43 | (24.2) | 0.153 |
| Prior AMI | 54 | (15.2) | 20 | (11.2) | 34 | (19.1) | **0.039** |
| Priorbleeding | 6 | (1.7) | 5 | (2.8) | 1 | (0.6) | 0.107 |
| Prior Stroke | 4 | (1.1) | 1 | (0.6) | 3 | (1.7) | 0.311 |
| PAD | 24 | (6.7) | 14 | (7.9) | 10 | (5.6) | 0.398 |
| Prior (30 days) major trauma or surgery | 10 | (2.8) | 2 | (1.1) | 8 | (4.5) | 0.054 |
| CKD | 76 | (21.3) | 41 | (23.0) | 35 | (19.7) | 0.438 |
| LVEF ≤30% | 29 | (8.1) | 14 | (7.9) | 15 | (8.4) | 0.846 |
| NIV | 16 | (4.5) | 6 | (3.4) | 10 | (5.6) | 0.306 |
| High-risk clinical profile | 56 | (15.7) | 29 | (16.3) | 27 | (15.2) | 0.771 |
| OTI | 29 | (8.1) | 16 | (9.0) | 13 | (7.3) | 0.561 |
| Inotropes | 33 | (9.3) | 19 | (10.7) | 14 | (7.9) | 0.361 |
| CCA | 37 | (10.4) | 19 | (10.7) | 18 | (10.1) | 0.862 |

Values are expressed as mean±SD or n (%).

STEMI, ST elevation myocardial infarction, CAD: coronary artery disease; PCI, percutaneous coronary intervention; CABG, coronary artery bypass grafting; AMI, acute myocardial infarction, PAD, peripheral artery disease; CKD; chronic kidney disease; OTI, orotracheal intubation; CCA, cardiocirculatory arrest; LVEF: left ventricular ejection fraction.
